# Treatment of Sciatica

**Published:** 1907-07

**Authors:** E. S. McKee

**Affiliations:** Cincinnati


### ABSTRACTS.

### Treatment of Sciatica.

By E. S. McKEE, M.D., Cincinnati.

THE following is taken from the *Monatschrift fuer Orthopaedische Chirurgie und physikalische Heil—und Untersuchungsmethoden,* No. 4, April, 1907, beilage zu *Deutsche Medizinal Zeitung,* 25 March, 1907, of Berlin, under the title Behandlung der Ischias von Dr. E. S. McKee, Cincinnati, and is abstracted from the original in *Blatter fuer Klinische Hydrotherapie,* February, 1907, of Vienna.

First a calomel purge, then salts; in constitutional disease as soon as possible introduce treatment for the cause, caution with morphine; in rheumatic cases, salicylate preparations; in syphilis, iodides; in gout, colchicum and saline mineral waters.

Hypodermic injection of sterilised air. First sterilise the immediate cutaneous region, then insert a sterilised Pavas needle into the skin and connect with a thin rubber tube, which is again connected with a glass tube containing a bit of sterilised cotton, and then one drives by means of a rubber balloon the air slowly under the skin. Naturally, care must be taken that the Pavas syringe needle be not run into a bloodvessel which easily occurs. Gentle massage should follow this procedure till the air is all absorbed. The rest cure of Wier Mitchell is in some cases very successful, especially in those where their means of livelihood has been an active one. Massage along the nerve though quite painful often breaks up adhesions, which are the cause of the disease in many instances. It is a rule however, that in true neuritis, massage is not followed by good results. Massage and especially mechanic vibrations are of special worth in chronic cases with beginning atrophy.

Hydrotherapy systematically used deserves due consideration. The wet pack over night diminishes the pain remarkably as well as having a favorable influence on the neurotic process. The next morning the leg is unpacked, washed with warm water and massaged. Ten or twelve of these applications are usually sufficient. In subacute cases the half steam bath is of service. Patient sits in a steam container which reaches only above the hips. This half steam bath does not weaken the patient and allows of a higher temperature; 44°C. can be endured for 10-15 minutes without discomfort. Afterward the patient is put in a bath of 35°C. for eight minutes and douches the diseased hips with water at 40 to 44°C. This procedure can also be combined with swimming baths and very often the drinking of large quantities of water is advisable.

Electrotherapy. Galvanic current direct on the nerves 4-8 minutes, highest 3 to 5 milliamperes. Also the interrupted galvanic current stimulates the muscles in a mild way, holds the tissues in a good condition and prevents atrophy. The static (Franklin) douche with positive pole on the painful part is followed by good results. The apparent anodyne action of faradism in sciatica is due to its alterant action on the muscular tissue and through that on the circulation. The current should not be increased to painfulness.

Surgical treatment.—In long standing cases one is often led to make an exploratory incision, lay the nerve bare, and separate some adhesions. In bloody nerve stretching, we find good and bad experiences. This operation is directly contraindicated where neuritis is present as in these cases myelitis not infrequently follows. There is also a so-called bloodless nerve stretching wherein the patient is under ether, the thigh is forcibly flexed on the hip-joint with the leg extended at the knee which position is maintained for some minutes.

The cure is easier when the patient is not too old and does not suffer from other diseases. The principle thing in the treatment consists in the careful observation of the patient, so that nothing is overlooked. The physician shall at once in the beginning commence with a systematic cure and if it does not go forward seek for his failure, for failure lays somewhere certainly. Exact diagnosis of the condition of the patient is the first and last means of treatment.

